# Regulation of highly homologous major urinary proteins in house mice quantified with label-free proteomic methods†
†Electronic supplementary information (ESI) available. See DOI: 10.1039/c6mb00278a



**DOI:** 10.1039/c6mb00278a

**Published:** 2016-07-18

**Authors:** Viktoria M. Enk, Christian Baumann, Michaela Thoß, Kenneth C. Luzynski, Ebrahim Razzazi-Fazeli, Dustin J. Penn

**Affiliations:** a VetCore-Facility for Research , University of Veterinary Medicine Vienna , Veterinärplatz 1 , A-1210-Vienna , Austria; b SCIEX Germany GmbH , Landwehrstraße 54 , D-64293 Darmstadt , Germany; c Department of Integrative Biology and Evolution , Konrad Lorenz Institute of Ethology , University of Veterinary Medicine Vienna , Savoyenstraße 1 , A-1160-Vienna , Austria . Email: Dustin.Penn@vetmetuni.ac.at

## Abstract

Major urinary proteins (MUPs) are highly homologous proteoforms that function in binding, transporting and releasing pheromones in house mice. The main analytical challenge for studying variation in MUPs, even for state-of-the-art proteomics techniques, is their high degree of amino acid sequence homology. In this study we used unique peptides for proteoform-specific identification. We applied different search engines (ProteinPilot™ *vs.* PEAKS®) and protein databases (MUP database *vs.* SwissProt + unreviewed MUPs), and found that proteoform identification is influenced by addressing background proteins (unregulated urinary proteins, non-MUPs) during the database search. High resolution Q-TOF mass spectrometry was used to identify and precisely quantify the regulation of MUP proteoforms in male mice that were reared in standard housing and then transferred to semi-natural enclosures (within-subject design). By using a designated MUP database we were able to distinguish 19 MUP proteoforms, with A2CEK6 (a Mup11 gene product) being the most abundant based on spectral intensities. We compared three different quantification strategies based on MS1- (from IDA and SWATH™ spectra) and MS2 (SWATH™) data, and the results of these methods were correlated. Furthermore, three data normalization methods were compared and we found that increased statistical significance of fold-changes can be achieved by normalization based on urinary protein concentrations. We show that male mice living in semi-natural enclosures significantly up-regulated some but not all MUPs (differential regulation), *e.g.*, A2ANT6, a *Mup*6 gene product, was upregulated between 9-fold (MS1) and 13-fold (MS2) using the designated MUP database. Finally, we show that 85 ± 7% of total MS intensity can be attributed to MUP-derived peptides, which supports the assumption that MUPs are the primary proteins in mouse urine. Our results provide new tools for assessing qualitative and quantitative variation of MUPs and suggest that male mice regulate the expression of specific MUP proteoforms, depending upon social conditions.

## Abstract 2

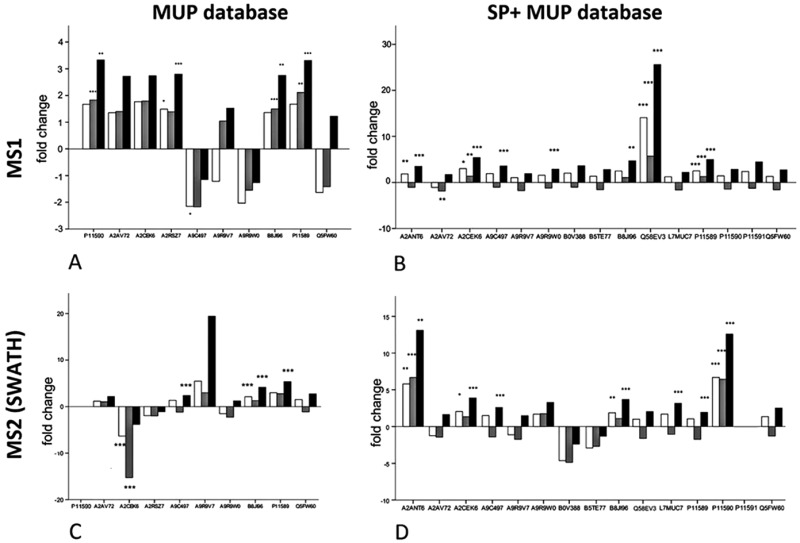
We performed isoform-specific MUP quantification on MS1 and MS2 level in response to increased social interaction of male wild house mice by seminatural housing.

## Introduction

Major urinary proteins (MUPs) mediate chemical communication in house mice by controlling the transport and release of volatile pheromones from urinary scent marks^1,2^ and serving as pheromones themselves.^3^ It has been suggested that mice express individually unique and consistent MUP patterns reflected by unique electrophoretic gel profiles, and thereby control individual and kin recognition.^4–6^ This ‘barcode hypothesis’ was recently tested in wild mice, but individual MUP profiles showed less individual variation and more fluctuation than previously assumed.^7^ Moreover, this hypothesis was based on the untested assumption that gel-based separation techniques reflect actual MUP protein variation.^7^ Recent studies have found that the amount of MUP proteins excreted in urine can be dynamically regulated, depending on nutritional status, health and social status (condition- and context-dependent MUP regulation).^8–11^ These studies measured total urinary protein concentration, which is widely assumed to reflect actual MUP excretion, or focused on the expression of one or few specific MUP genes. No studies to our knowledge have tested whether MUPs are differentially regulated. Our general aims were to compare different gel-free and label-free proteomic techniques (MS1 and MS2 using SWATH) and use these methods to quantify changes of proteoform-specific MUP-regulation in wild-derived house mice (*Mus musculus musculus*). We here present analytical strategies using high resolution mass spectrometry to quantify proteoform specific MUP expression and thus reflect inter and intra-individual MUP variation.

**Fig. 1:**
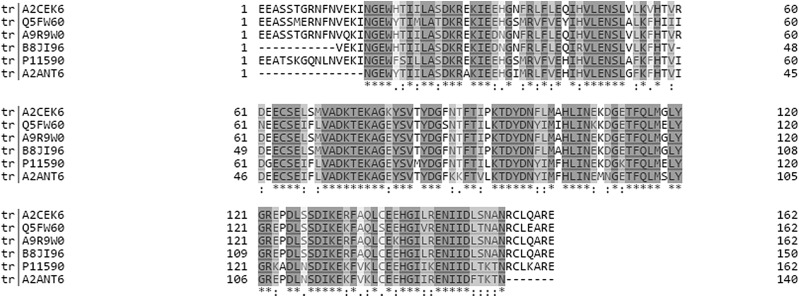
Multiple sequence alignment of six MUPs that were shown to be significantly upregulated regardless of the quantification strategy (see Fig. 4). The alignment was created using Clustal Omega on Uniprot. Grey shading shows 100% homology and white indicates positions that are dissimilar between proteoforms. It should be noted that the sequences shown represent only a subset of MUPs identified in our study and thus fewer unique peptides exist for the respective proteoforms when all available sequences are aligned.

The main technical challenge for measuring variation in MUPs is due to their remarkably high homology at genetic and protein levels (see Fig. 1). In house mice, MUPs are encoded by at least 21 *Mup* loci that are closely linked within a large cluster containing central (>97% homology) and peripheral (*Mup* 3, 4, 6, 20, and 21; 82–94% homology on c-DNA level) MUPs, which are easier to distinguish.^12–14^ The analytical challenge is discriminating individual MUP family members (up to 34 different protein sequences are currently published in Uniprot; wild mice may have more). Conventional antibody-mediated methods and genetic analyses cannot distinguish individual MUPs, due to their homology on gene and protein levels.^2,15,16^ Thus, MUPs represent a difficult and challenging analytical problem, even for state-of-the-art proteomic techniques.

Previous studies on MUP variation have mainly relied on gel-based methods, but protein quantification approaches are increasingly moving away from (2-dimensional) gel based methods.^17^ The barcode hypothesis, for example, is based on the untested assumption that gel-based separation techniques reflect actual MUP gene and protein variation.^4,7^ However, neither narrow-range IPG nor 2-dimensional electrophoresis strategies are capable of fully resolving different MUPs, as high-resolution mass spectrometry (Q-TOF MS) revealed more than one MUP per band/spot (Thoß *et al.* unpublished). Mass spectrometry studies using labeled proteins to quantify intact MUPs were unable to quantify changes of central MUPs.^18^ Due to their high homogeneity that impairs chromatographic separation, deconvolution-assisted top-down analysis of intact MUP–proteoform mixtures were likewise unable to discriminate individual MUP family members.^19^ Therefore, bottom-up high-resolution mass spectrometry is required for identifying and quantifying proteoform specific changes of MUP-profiles,^20^ though this is a challenging problem that even pushes bottom-up proteomics to its limits.^21,22^

Our aims were to apply state-of-the-art proteomic techniques to measure qualitative and quantitative variation in MUPs. We used a within-subject design to quantify the differential expression of urinary MUPs by male house mice living in different conditions. Social interactions in the laboratory have been found to affect the regulation of urinary MUPs.^8,11^ However, studies are needed to determine whether MUP production is socially regulated in more natural conditions (ecological validation). There are increasing studies showing changes in gene expression due to individual *versus* group housing.^23^ Furthermore, determining how mice regulate MUP production has important implications for understanding the functions of regulating MUP production (*e.g.*, differential regulation of specific MUPs is inconsistent with the barcode hypothesis). Therefore, we profiled changes in urinary MUPs associated with changing housing and social conditions. We analyzed urine samples from male mice first collected in standard colony conditions, and then collected from the same mice while living in semi-natural social conditions. We applied a gel-free, label-free, shotgun MS-based strategy for identification and subsequent proteoform-specific quantification of the exceptionally homologous MUP proteins. Previous studies on quantification of similarly homologous protein superfamilies, such as cytochrome P450 enzyme family, kinesins, dyneins, chaperones (*e.g.* Hsp70), amyloid precursors, and tubulin, show that these proteins have highly similar tryptic efficiencies and fragments. Consequently, proteoform-specific unique peptides are then used for MS-based quantification.^24^ We adopted this proteomics strategy to measure temporal dynamics in urinary MUPs as a model system for studying quantitative MUP regulation. For this approach, high-resolution mass spectrometry is required that allows mass accuracies <2 ppm RMS^25^ and detects single amino acid differences between MUP proteoforms. Moreover, the instrument's high sensitivity allows quantifying MUPs expressed even in low abundance. We employed two methods of label-free protein quantification: MS1 to compare unique peptide precursor ion intensities of the respective proteins; and MS2 (using Sequential Window Acquisition of All Theoretical Fragment Ion Mass Spectra or SWATH™) to compare the number of fragment spectra identifying unique peptides.^26–28^ SWATH™-based quantification is well-suited for untargeted quantitative analysis (*e.g.*, unreviewed MUPs) because it fragments all peptides present in the sample.^29,30^

Due to the high homology of MUP proteoforms, sequence information is scarce on gene and protein level. Current databases list only 9 reviewed MUPs, although unreviewed or putative MUPs (c-DNA derived sequences in UniProt) also exist. These proteoforms often differ by a single amino acid only. Consequently, for mass spectrometry only proteoform-specific peptides can be used for protein identification and quantification. Even with high resolution MS, isobaric peptides can only be distinguished if fragment spectra are recorded (MS2).^27^ In general, relative protein quantification is computed from up- or down-regulation of low abundant analytes in a high abundant, constant matrix.^31,32^ Because MUPs are the main components of mouse urine, up-regulation of these also increases urinary protein concentrations. Therefore, selection of an appropriate normalization strategy is crucial which is furthermore influenced by identification of constantly expressed proteins (non-MUPs) addressed by the underlying database search.

To measure the regulation of MUPs by male house mice, we applied different methods – MS1 and MS2 based Q-TOF mass spectrometry – with aim to perform proteoform-specific MUP identification and quantification. We also investigated the following methodological questions:

(1) How can we use available c-DNA derived sequence information to assess comprehensive urinary MUP diversity and validate putative proteoforms on protein level?

(2) How is proteoform-specific MUP identification influenced by using different databases and search engines?

(3) How can mass spectrometric approaches (MS1 and MS2) quantify differential expression of MUPs?

(4) How do normalization strategies influence the results of quantitative proteomics?

(5) How do the aforementioned databases influence MUP quantification?

(6) How do fold-changes computed by MS1 or MS2 quantification correlate?

(7) What proportion of total urinary proteins is comprised of MUPs and which proteoforms are most abundant (relative composition)?

## Experimental procedures

### Materials and methods

#### Animal housing

Animals were F1 offspring of wild-caught house mice (*Mus musculus musculus*) trapped and bred at the Konrad Lorenz Institute of Ethology (Vienna, Austria). They were housed individually in standard housing (type IIL cages, 36.5 × 20.5 × 14 cm, Tecniplast, Germany) and then introduced to semi-natural enclosures (four 3.4 × 4 m environmentally enriched indoor enclosures with 4 male and 4 female mice in each) where they lived in more natural conditions. Food (Altromin rodent diet 1324) and water were provided *ad libitum* and temperature was maintained at 22 ± 2 °C. Mice were kept on a 12 : 12 h light : dark cycle. At the start of the experiment, animals were three to six months old. Further details can be found elsewhere.^7^

Our procedures were in accordance with ethical standards and guidelines in the care and use of experimental animals of the Ethical and Animal Welfare Commission of the University of Veterinary Medicine Vienna (Permit No. 02/08/97/2013).

#### Urine sampling and measurement of protein concentrations

All urine samples used for shotgun proteomics were collected from each mouse at 4-week intervals using metabolic cages (Tecniplast, Germany). Immediately following the second sample period, mice were released and remained in a semi-natural enclosure for 3 subsequent samplings (total housing duration in social conditions was 12 weeks). Metabolic cages were employed for urine collection because they mitigate contamination by fecal boli and reduce handling stress. Mice were promptly released from metabolic cages after >70 μL of urine had been collected. Collection times never exceeded 90 min and thus urinary MUPs are not expected to be influenced by handling stress.^33^ Furthermore, the sampling procedure was identical at t1 and t2 and therefore cannot account for the observed differences. Collected urine was immediately transferred to Eppendorf tubes and stored at –80 °C until further use. Protein concentrations were measured using a standard Bradford assay^34^ on a 96-well microplate. Triplicates were analyzed and the measurement was repeated if values were outside a range of ±10%.

13 male mice were investigated at two time points (26 urine samples) to compare quantification strategies. The average protein concentration of these animals was 2.0 μg μL^–1^ while the average of individuals was 2.6 μg μL^–1^ during and 1.4 μg μL^–1^ before housing in semi-natural enclosures. To account for this difference of urinary protein concentrations, quantification results were normalized by protein concentrations (manual scale factors). A paired design was established by creating 2 urine pools for each mouse (*n* = 26): a pool of the first 2 samples obtained before enclosure housing (t1) and a pool from the 3 samples obtained during seminatural housing (t2). Urine was pooled to increase the amount of urine and to account for daily fluctuations of urinary protein concentrations (*e.g.* influenced by water uptake). Furthermore, urine of 23 additional male mice was sampled as outlined above and used for confirmation of putative MUP sequences transcribed from c-DNA. The assumption that MUP profiles remain stable over time has recently been challenged^35^ and we determined which MUPs are monomorphic, common, rare or not expressed in this population.

#### Protein digestion

For proteomic analysis aliquots of 2 μL were adjusted to 10 μL using 50 mM Tris(hydroxymethyl)aminomethane hydrochloride (Tris–HCl) and denatured (10 μL 8 M urea in 50 mM Tris–HCl) before reduction with dithiothreitol (DTT) (2 μL 50 mM DTT in 25 mM ammonium bicarbonate ABC) at 60 °C for 30 minutes. Alkylation was performed for 45 minutes in the dark (6 μL 55 mM iodoacetamide IAA in 25 mM ABC). Residual IAA was reduced using DTT as described above. Trypsin working solution was added in a 1 : 50 ratio (trypsin : protein) depending on the respective protein concentration. The volume was adjusted to 50 μL using 25 mM ABC. The digestion was performed at 37 °C for 8 hours and stopped by acidification with trifluoroacetic acid to a final concentration of 0.5%.

#### Peptide separation

Following digestion, peptides were separated by a nano high performance liquid chromatography system (Ultimate 3000 RSLC) using a pre-concentration trap column (Acclaim® PepMap™ μ-Precolumn) and a nano separation column (Acclaim® PepMap™ RSLC 75 μm × 25 cm, nano Viper C18, 2 μm, 100 Å) (both Dionex™, Thermo Fisher Scientific, USA). The mobile phases used were water (LC/MS grade Fisher Scientific, USA) with 0.1% formic acid (A) and 80% acetonitrile (ACN) with 0.1% formic acid (B). A mobile phase gradient from 4% B to 35% B in 120 min and then up to 90% B in 15 min followed by a washing step with 90% B for 10 min was run.

#### Mass spectrometry – description of MS1 and MS2 modus

The mass spectrometer used was a quadrupole time of flight system (Q-TOF-MS, TripleTOF™ 5600+, Sciex, USA) coupled online to the LC with an ESI nano source. Data were recorded from *m*/*z* 250 to 1500 to obtain high peptide coverage and include short unique peptides. Data acquisition and interpretation were performed using Analyst® TF 1.7, ProteinPilot™ 5.0 (both Sciex, USA) and Dionex™ Chromeleon™ 6.8 (Thermo Fisher Scientific, USA).

Label-free quantification was performed based on proteoform specific peptide intensities (MS1) and fragments created from these (SWATH™). As both strategies are untargeted, it is possible to analyze a single dataset with different databases or ion libraries to compare the influence of the databases used.^36,37^ Run–run alignment was performed using either approach. For MS1 quantification, mass lists (XIC lists) of proteotypic peptides identified in all samples investigated served as a basis for quantification. These peptide masses were then used to extract MS1 quantitative information from individual mass spectra (IDA and SWATH™). Similarly, SWATH™ quantification was based on an ion library created from an identification run that also contains unique peptides of combined mass spectra.

SWATH™ is a method for peptide based quantification showing comparable reproducibility with targeted MRM as shown in other studies.^38^ Our study design was performed in accordance with the German Network for Bioinformatics Infrastructure (NBI). Pooled urine of individuals at t1 and t2 was analyzed in a pilot study and showed high technical reproducibility (see Fig. S1, ESI†). Therefore, the SWATH experiment was conducted using single measurements of urine samples from the 13 individuals at t1 and t2.

### Data processing and analysis

#### MUP identification

Proteins were identified in a bottom-up approach from measured peptides utilizing ProteinPilot™ 5.0 (PP) with the inbuilt Paragon algorithm as well as PEAKS® Studio 7 (BSI, Canada) using a SPIDER search. For each search algorithm, two different databases were compared: a designated MUP-database (MUP DB) and all murine proteins from SwissProt (SP) plus unreviewed MUPs (SP+MUP DB). Protein sequences were downloaded from Uniprot for creation of the databases used in this study and manually cleared of redundancies those partially originate from differences in signal peptides cleaved prior to urinary secretion. The MUPs present in the two databases were identical and other proteins were included to investigate the influence of constantly expressed urinary housekeeping proteins on MUP quantification. 16 773 proteins were contained in the SP+MUP (Swissprot+unreviewed MUPs) database and 32 of these were MUPs. If taken alone, these 32 MUPs made up the MUP database. We refer to these highly homologous MUP family members encoded by the same gene cluster as ‘proteoforms’, as this term is commonly used to describe differences due to genetic sequence variation.^39^

**Fig. 2:**
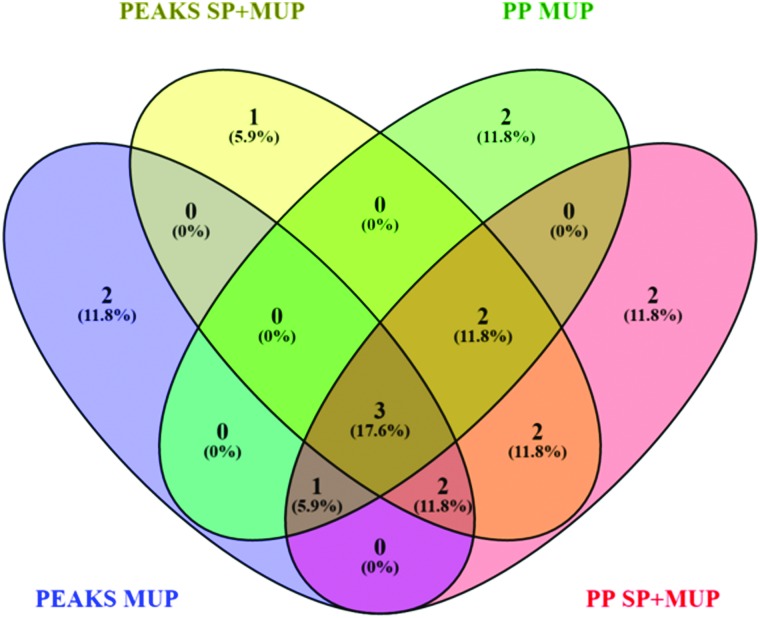
Venn diagram^48^ showing differences in proteoform identification depending on databases (MUP *vs.* SP+MUP DB) and search algorithms (PEAKS® *vs.* ProteinPilot™). Identification results were generated using all 36 individually analyzed urine samples (sampled at t1 and t2 regardless of social status) combined for each database search.

A false discovery rate (FDR) cutoff of 1% was used for all strategies to ensure high confidence protein identifications. Although we commonly use a minimum of two peptides to consider protein identifications statistically significant, we had to allow identifications with one proteotypic peptide due to the high homology of MUPs. Identification by the different algorithms was based on the proteotypic peptides identified in a combined dataset of all 36 identification runs using either PEAKS® or PP (Fig. 2). 36 individual ProteinPilot™ searches in the MUP database were used to classify high and low abundant proteoforms. Frequencies were calculated by dividing the number of individuals expressing a proteoform through the total sample size. As outlined above we used a total of 36 individuals here, 18 of which were sampled at two time points.

Due to differences in protein identification we found from utilizing different search-engines, assessment of MUP variety and creation of SWATH™/MS1 ion libraries were performed based only on PP-searches. We used a subset of animals (*n* = 13) for testing quantitative changes of MUP profiles at two time points.

#### MUP quantification

Proteotypic peptides of combined identification runs (*n* = 36) in ProteinPilot™ were used to create an ion library for relative quantification using PeakView®. MS1 quantification was performed by extraction of precursor mass peak areas using MasterView™ in PeakView®. For MS2 based quantification, a data-independent approach (SWATH™, Sciex, USA) based on peptide fragment masses was used. Combination of both approaches results in enhanced specificity and data robustness compared to quantification on MS1/MS2 level alone.^40^ To achieve maximum comparability between protein identification, as well as MS1 and MS2 quantification, further MUP identification was performed using the same software (ProteinPilot™).

#### Normalization strategies

We examined how normalization can be performed in a quantitative proteomics study, in which upregulation of the quantification targets (MUP proteoforms) induces a change of overall protein concentration. Three different normalization strategies were compared in MarkerView™ 1.2.1 (Sciex, USA): (1) no normalization was used to reflect the changes of total protein concentrations on MS-level. (2) Total Area Sums (TAS) was chosen to normalize based on MS1-intensities. (3) Manual scale factors were used to normalize MS signals based on different protein concentrations and to account for ionization efficiencies of individual peptides.

Alternatively, manual scale factors (Bradford protein concentrations of urinary samples) can be used to normalize on the protein content of each individual sample. The three different approaches were chosen to retain information regarding total protein concentration on MS-level because prior studies^41–43^ have found that urinary protein concentration itself (predominantly composed of MUPs) serves as relevant biological information. To show relative differences of protein composition, protein concentrations were chosen for normalization. As total ion chromatograms and urinary protein concentrations did not perfectly correlate, however, TAS was also considered to account for any other factors influencing Bradford measurements.

#### Correlation of quantification strategies

Quantification on both MS1 and MS2 level and comparing the respective results provides an estimation of robustness of the used techniques. MS1 quantification can be performed using different software packages^44^ including PEAKS®, MasterView™ in PeakView® and Skyline. Here, we chose data processing with PeakView® and Skyline which allow run–run alignment through using an ion library. MS1 Data processing from IDA spectra was performed using MasterView™ in PeakView®. The SWATH™ 2.0-plugin in PeakView® was used to extract MS2 data from SWATH™ spectra. Additionally, MS1 data were extracted from SWATH™ spectra using Skyline and were also used for MS1 quantification. Proteins that were regulated < two-fold or had a *p* > 0.05 were considered non-significant and thus excluded. We accounted for multiple testing using Bonferroni–Dunn correction.^45,46^

#### MUP proteoform composition

For calculating relative MUP proteoform compositions, areas of proteoform-specific peptides were extracted from SWATH™ data. Equal ionization efficiencies of MUP-derived peptides were assumed due to their high homology in order to use a 100% method for relative quantification. Normalization was performed according to the number of unique peptides per MUP quantified. To estimate the relative urinary composition, MS intensities of MUP-derived peptides were compared to other urinary proteins. Here, extracted areas of all MUP-specific peptides (unique and shared between proteoforms) were summed up and divided by the total intensity of all quantified proteins.

### Statistical analysis

To compare MUPs expressed in different housing conditions (standard cages *vs.* semi-natural conditions), paired *t*-tests based on the calculated peak areas were performed using MarkerView™ for MS1 and MS2 quantification approaches. Data were normalized according to MS-intensities (TAS) and protein concentrations (manual scale factors) of urine samples. Figures were created using SPSS Statistics for Windows (Version 22.0. IBM Corp., USA.), if not stated otherwise. All *p*-values computed in MarkerView™ were adjusted for multiple testing using Bonferroni–Dunn correction.^45,46^ The respective *p*-values depend on the number of quantified MUP proteoforms and are indicated as asterisks and explained in figure captions.

## Results

### MUP identification

#### Proteoform specific identification of unreviewed MUPs

We analyzed qualitative variation within and between individuals in excreted MUPs (Table 1). We detected 19 different MUP proteoforms expressed in urine (encoded by 14/21 *Mup* loci, Table 1). Many of these have only been described so far on gene-level^2^ and we could now detect them on protein level. Two proteoforms were ubiquitous (100% of mice) in this population, four were common (50–70%), seven were uncommon (30–49%) and six were rare (<10%). Although we detected different MUPs expressed in different individuals, all mice expressed 8–12 different MUP proteoforms per individual. Some MUPs (Q5FW60 – *Mup*20 and B8JI96 – *Mup*14) were identified in all samples analyzed. Our results indicate that the mice regulated the type of MUP they excreted, rather than the number of MUPs. This finding indicates that regulation mechanisms control MUP expression (*e.g.*, complex hormonal control or different promoter genes).^47^ An example is *Mup*1 whose products A2CEL1 and Q58EV3 differ both in sequence and abundance.

**Table 1:** MUP isoforms detected in wild mice, showing the corresponding genetic locus and frequency of occurrence (*n* = 36). Protein identification results of 36 individuals, 18 of which were sampled at two different time points are included in order to show a comprehensive summary of urinary MUP isoforms and their identification frequency. MS data were analyzed using ProteinPilot™ with the Paragon algorithm. The MUP database was chosen and a minimum peptide confidence of 95% was used alongside with an FDR of 1%. MUPs were considered identified if MS and MS/MS spectra for proteotypic peptides could be matched with database entries

Proteoform	Gene	Frequency of proteoform-specification
tr|B8JI96	*Mup*14	100%
tr|Q5FW60	*Mup*20	100%
tr|A2AV72	*Mup*6	70%
tr|A9R9V7	*Mup*21	59%
tr|P11590	*Mup*4	54%
tr|B5TE76	*Mup*17	50%
tr|A9C497	*Mup*19	48%
tr|A2CEL1	*Mup*1	44%
tr|P11589	*Mup*2	41%
tr|A9R9W0	*Mup*15	41%
tr|A2CEK6	*Mup*13	39%
tr|B5X0G2	*Mup*17	35%
tr|P11591	*Mup*5	30%
tr|A2ANT6	*Mup*6	9%
tr|Q3KQQ2	*Mup*3	7%
tr|Q80YX8	*Mup*21	7%
tr|P04939	*Mup*3	6%
tr|Q58EV3	*Mup*1	2%
tr|L7MUC7	*Mup*7	2%

#### Effects of databases and search engines on MUP identification

The effects of different search parameters on the identification of MUPs are shown in Fig. 2. For evaluating the reliability of protein quantification with minor sequence differences, an unreviewed database based on UniProt was chosen. It contained all 34 MUP proteoforms distinguishable by unique peptides. This database showed intrinsically higher homology within its entries than the reviewed MUP entries in SwissProt. In order to show the effects of constantly expressed background proteins (non-MUPs) on identification algorithms, unreviewed MUP proteoform sequences were also added to a database of reviewed mouse proteins (SwissProt).

Additionally, two different search algorithms, PEAKS® and ProteinPilot™ were compared (Fig. 2). It is clearly shown that the databases and search algorithms used for identification of MUPs play a crucial role regarding proteoform specific identification of central MUPs (higher homology). Based on the same dataset (all 36 runs combined) some MUPs were uniquely identified using PEAKS® or ProteinPilot™. We could also observe differences within the same software depending on whether a pure MUP database (MUP DB) or SwissProt sequences and unreviewed MUPs (SP+MUP DB) were used.

As shown in Fig. 2, three proteins were identified regardless of the search parameters and algorithms used: A9C497 (*Mup*19), Q5FW60 (*Mup*20) and A9R9V7 (*Mup*21). Although A9C497 (*Mup*19) is classified a central MUP, it is located at the outer edge of the *Mup*-cluster and thus slightly more different from other central MUPs. Q5FW60 (*Mup*20) and A9R9V7 (*Mup*21) are both classified as peripheral MUPs showing lower homology. The three aforementioned MUPs have an average pairwise sequence identity of 71% compared to central MUP's homology often exceeding 97%. Variation regarding identification of central MUPs is high.

As shown in Fig. 2, the proteoforms exclusively identified by one of the aforementioned databases are listed below:

PEAKS® MUP (blue): A2RSZ7 (*Mup*5) and A2CEL1 (*Mup*1)

PEAKS® SP+MUP DB (yellow): Q80YX8 (*Mup*21)

PP MUP (green): B5TE76 (*Mup*14) and P11590 (*Mup*4)

PP SP+MUP (red): P02762 (*Mup*6) and A2CEK6 (*Mup*13)

Following analysis of protein identification, we next conducted further quantitative analyses to examine the relative expression of different proteoforms, and how they changed over time and across housing treatments.

### MUP quantification

To examine the response of MUP proteoforms to sociality we quantified relative expression of urine collected pre-social conditions (t1) and during social conditions in a semi-natural enclosure. Expecting an increase in expression under social conditions at t2, Student's *t*-tests on paired data was used to compare relative MUP proteoform expression at each time point (Fig. 2). Furthermore, we compared two quantification strategies (MS1 and MS2) and two databases of different complexity. This approach allowed us to compare MUP proteoform expression in response to sociality across varying bioinformatic methods.

**Fig. 3:**
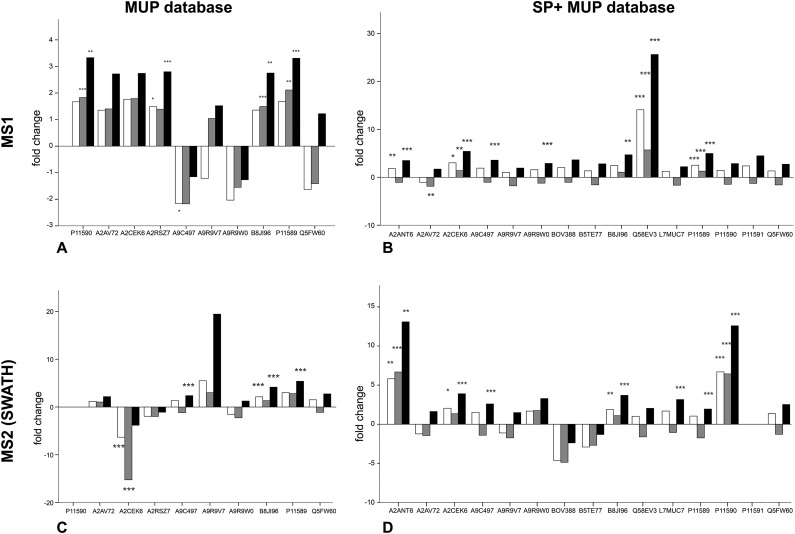
Regulation of different MUP proteoforms across housing conditions, comparing different quantitative approaches (MS1 and MS2) and databases (MUP DB and SP+MUP DB) to show effects of background normalization on relative quantification of specific proteoforms. White bars = no normalization, grey bars = TAS normalization, black bars = manual normalization to protein concentration. Adjusted *p*-values depend on the number of proteins quantified and are indicated as asterisks: (A) adjusted *p* value (correcting for 10 tests): 0.005, * = *p* < 0.005, ** = *p* < 0.002, *** = *p* < 0.001; (B) adjusted *p* value (correcting for 15 tests): 0.0033, * = *p* < 0.003, ** = *p* < 0.002, *** = *p* < 0.001; (C) adjusted *p* value (correcting for 9 tests): 0.006, ** = *p* < 0.002, *** = *p* < 0.001; (D) adjusted *p* value (correcting for 14 tests): 0.0036, * = *p* < 0.003, ** = *p* < 0.002, *** = *p* < 0.001.

We investigated changes in MUP expression in house mice kept in conventional cages (t1) and later in large, seminatural conditions (t2) (Fig. 3). Based on previous studies, we expected changes in MUP expression due to social and sexual interactions.^11^ We quantified relative MUP expression of different proteoforms across housing conditions by mass spectrometry and found significant upregulation of specific MUPs. In addition, we compared two quantification strategies (MS1 and MS2) and two databases of different complexity with three normalization strategies each.

**Fig. 4:**
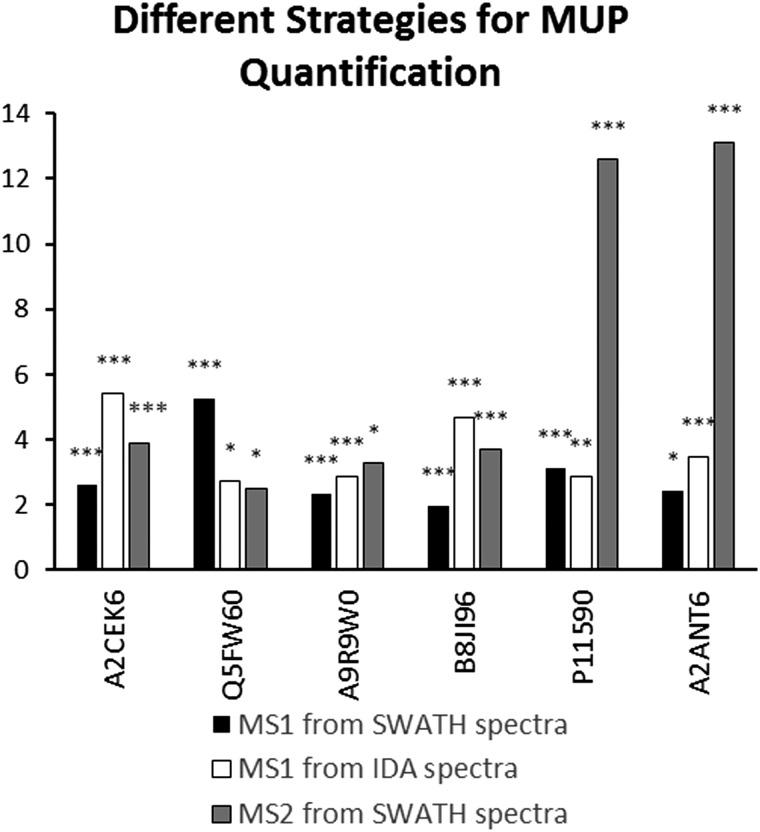
MUP upregulation (*p* < 0.05 fold-change >2) through semi-natural housing (t2) computed by different MUP quantification strategies. Proteins were identified using SP+MUP database and normalized using manual scale factors. Significance levels (Bonferroni–Dunn-corrected for 6 tests) are indicated by asterisks: * = *p* < 0.008, ** = p < 0.003, *** = p < 0.002.

Upregulation could be shown on both MS1 and MS2 level (for correlation see Fig. 4). Using different databases (Fig. 3(B) and (D)) more MUPs could be quantified when the entire urinary proteome (including unreviewed MUP proteoforms) was addressed by the underlying database search. Using the comprehensive database instead of a designated MUP database could improve quantification from 10 to 15 proteoforms in MS1-quantification and from 9 to 14 proteoforms on MS2 level. B8JI96 (*Mup*14) and P11589 (*Mup*2) were significantly upregulated when normalizing by protein concentration in MS1 and MS2 modus regardless of the database used.

### MS1 quantification

When MS1 quantification was performed using the dedicated MUP database, P11590 (*Mup*4), A2RSZ7 (*Mup*5), B8JI96 (*Mup*14) and P11589 (*Mup*2) were significantly (*p* < 0.002) upregulated by housing in semi-natural enclosures (Fig. 3(A)). Using a comprehensive database (SP+MUP DB) the differences between normalization strategies were more evident (Fig. 3(B)). We found that TAS normalization had an enormous effect on the calculated protein regulations and their corresponding statistical significance. This is due to the low complexity of the murine urinary proteome, which is mainly composed of MUPs. As MUP upregulation influences the total protein concentration, it needs to be considered for data normalization. Therefore, the main focus of our interpretation was based on manual normalization with total protein concentration (black bars). Using the SP+MUP database with normalization on protein concentration, A2CEK6 (*Mup*13), A9R9W0 (*Mup*15), P11589 (*Mup*2), Q58EV3 (*Mup*1), A9C497 (*Mup*19) and A2ANT6 (*Mup*6) were significantly upregulated during semi-natural housing (t2) compared to conventional housing (t1) (*p* < 0.001). None of the MUP proteoforms we detected were significantly downregulated.

### MS2 quantification (SWATH™)

**Fig. 5:**
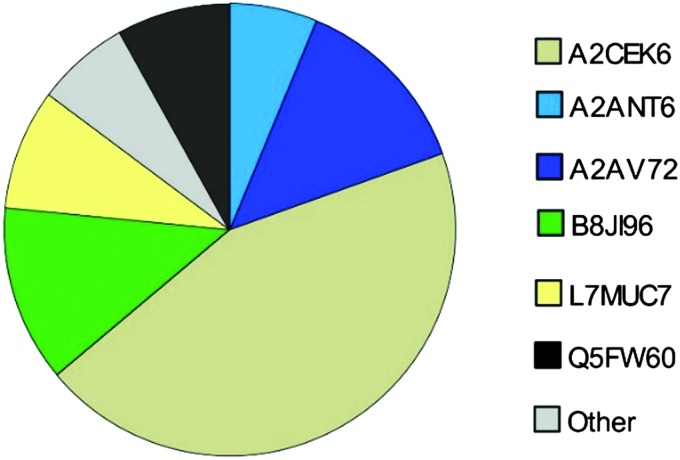
MUPs Composition based on SWATH™ quantification (*n* = 36) of urine using SP+unreviewed MUP as a database. Proteoform-specific peptide peak areas were normalized by the number of unique peptides per MUP to increase comparability. Low abundant MUPs (<5%) are classified as “Others” and marked in grey.

By normalizing MS2-data based on total protein concentration, we noted an upregulation (*p* < 0.001) of the following three proteoforms: A9C497 (*Mup*19), B8JI96 (*Mup*14) and P11589 (*Mup*2) regardless of the database used (Fig. 3(C) and (D)). Using the MUP database A2CEK6 (*Mup*13) appeared significantly downregulated without normalization or using normalization on TAS. As it is the most abundant MUP (see Fig. 5), the downregulation observed was not significant using normalization of protein concentration. This result exemplifies the analytical challenge arising when relative MUP quantification is performed with a changing background. Thus, the apparent downregulation of A2CEK6 (*Mup*13) using the MUP database is induced by relative upregulation of other proteoforms in response to semi-natural housing and is therefore non-significant when normalizing by urinary protein concentrations. As already observed in the MS1 approach, additional MUPs could be quantified using the comprehensive database on MS2 level and here we show that A2ANT6 (*Mup*6), A2CEK6 (*Mup*13), A9C497 (*Mup*19), B8JI96 (*Mup*14), L7MUC7 (*Mup*7), P11589 (*Mup*2), and P11590 (*Mup*4) were significantly (*p* < 0.002) upregulated (Fig. 3(D)).

### Correlation between MS1 (from IDA and SWATH™ spectra) and MS2 (SWATH™) quantification

We found high consistency between MS1 and MS2 data using the aforementioned approach. Consistent upregulation of specific MUPs was shown with background normalization using the SP+MUP database. We have chosen fold changes computed from MS1 (from IDA and SWATH spectra) and MS2 data for assessing the reproducibility of quantification strategies. Generally, upregulation of MUPs during semi-natural housing was similar between different quantification strategies. Two exceptions were P11590 (*Mup*4) and A2ANT6 (*Mup*6), which showed a higher upregulation using SWATH quantification than using MS1 based approaches.

### MUP proteoform composition

Here, we investigated the relative composition of urinary MUP proteoforms using a SWATH™ approach. Although SWATH™ is not used for absolute quantification, we can differentiate between MUPs showing low and high abundance (summarized as others) based on relative differences between peak areas of unique peptides and the number of unique peptides per proteoform.

We show, that the MUP proteoforms contributing highest relative unique peptide intensities are A2CEK6 (*Mup*13), B8JI96 (*Mup*14), A2AV72 (*Mup*6), Q5FW60 (*Mup*20), A2ANT6 (*Mup*6) and L7MUC7 (*Mup*7). Additionally, we confirmed the assumption that MUPs account for more than 90% of urinary proteins in mice^35,49^ by showing that MUP-derived peptides account for 85 ± 7% of MS-intensity.

## Discussion

In this study, we performed proteoform-specific MUP identification using high-resolution mass spectrometry and label-free quantification methods. The highly homologous nature of MUP proteoforms poses a challenging problem for qualitative and quantitative analyses of MUPs. Two quantitative approaches, MS1 and MS2, were evaluated using three different normalization strategies and two databases. Using a more comprehensive database (which includes non-MUP proteins but contained no new MUP sequences) increased the number of MUPs identified and quantified. We found that our results using MS1- and MS2-based quantification approaches were generally consistent, indicating technical reproducibility. The differences between using these methods for MUP identification and quantification are addressed below. We identified 19 different MUPs and all mice expressed 8–12 different MUP proteoforms per individual. The mice showed upregulation of some but not all MUPs after being transferred from standard cages (t1) to more natural social conditions (t2). We also provide evidence based on relative spectral intensities that MUPs comprise the most common proteins in urine of wild house mice. Below we address these and our other main findings in more detail.

MUPs of male house mice were identified to compare different databases (designated MUP database *vs.* SP+MUP) and search engines (ProteinPilot™ and PEAKS®). Because proteoform identification results differed between ProteinPilot™ and PEAKS®, we chose ProteinPilot™ for further data processing (*e.g.* calculating frequencies of proteoform-specific identification) and creation of ion libraries to ensure comparability with subsequent quantitative analyses. Our findings show that gene products of *Mup*14 (B8JI96) and *Mup*20 (Q5FW60-‘darcin’) are completely monomorphic. On the other hand, we detected more urinary MUP-proteins than found in previous studies, including some that have been not known to be excreted in urine (*e.g. Mup*4 products).^50,51^ However, detection of these proteoforms does not indicate their quantitative expression, and it is unclear if they are expressed at levels sufficient to influence olfactory detection. Our results thus provide evidence for individual variation in MUP protein proteoforms in wild mice, and we are comparing inter- *versus* intra-individual variation (MUP fingerprinting)^7^ in another study. As we explain in the next section, our results here also show that quantitative MUP proteoform expression shows differential and surprisingly dynamic changes across different social conditions.

To investigate MUP regulation, we used both an MS1- and an MS2-based quantification method. We analyzed urine samples from male mice to compare MUPs expressed in standard housing (mouse cages) *versus* seminatural conditions, where males experienced sexual and competitive social interactions. We found males showed a significant upregulation of some but not all MUPs, and identified a proteoform-specific pattern of quantitative changes. We found two MUPs, B8JI96 (*Mup*14) and P11589 (*Mup*2), were significantly upregulated using either database in MS1 and MS2 modus. For the first time, we present a strategy to measure different MUP proteoforms by monitoring proteotypic peptides and have thereby achieved substantial methodological improvement that allows for regulation analysis of specific MUP proteoforms.

The normalization strategy used influenced the results of MUP quantification (see Fig. 3). When normalizing using protein concentration, we attempted to account for the quantitative change of urinary proteins to investigate the regulation of individual proteoforms. It is assumed that normalisation by manual scale factors (protein concentrations) and TAS accounts for different protein concentrations while data without normalization also shows proteins that are upregulated from t1 to t2 but do not have higher intensities relative to other MUPs at t2. In contrast to normalization with protein concentrations, TAS (total area sums) normalization accounts for ionization efficiencies of individual peptides. Consequently, we showed that TAS normalization differs from adjusting quantification results using protein concentrations, which is not unexpected.

The size of the database is also critically important for data normalization, and especially for normalizing by TAS. We found that alignment of total protein concentrations resulted in most significant results. However, differences between normalization strategies were smaller than these between t1 and t2 and considered statistically non-significant (<2-fold regulated). We also found that the use of a comprehensive database (SP+MUP) increased the number of MUPs identified – even though it contains the same MUP sequences as the smaller database – and that fold changes could be computed at higher significance levels when MS intensities were normalized on protein content. Including constantly expressed urinary proteins (background normalization) in this process enabled us to quantify more MUPs in response to social changes. To explain why MUP regulation is altered by the use of SP+MUP compared to a designated MUP database, we have to consider that each MUP has only one to few proteotypic (proteoform-specific) peptides. These sequences only occur once in a given search database upon *in silico* digestion with the respective enzyme. Hence, when increasing database complexity, the likelihood that peptides, and especially short ones, remain proteotypic decreases. Thus, different database searches may result in different proteotypic peptides to be chosen for subsequent quantification. This effect can also explain the differences between fold changes shown in Fig. 3(A/B) or (C/D).

The high uncertainty of *Mup*-cluster sequencing approaches results in entries without evidence on protein level in UniProt, and here we show how high resolution mass spectrometry data can be used to distinguish products of genes that are otherwise indistinguishable. Unreviewed MUP sequences retrieved from Uniprot contain some 235 AA c-DNA transcripts. To test whether these putative long MUP proteoforms actually exist in mouse urine or whether they are cleaved prior to secretion, they were included in the MUP-database to enable screening for proteotypic peptides of these. However, no peptides could be mapped with these sequences, therefore we find no evidence that the longer forms are expressed on protein level. Thus, we used databases without these 235 AA MUP sequences for creation of ion libraries.

(Label free) quantification of MUPs is challenging due to their high degree of homology. Therefore, we evaluated the correlation between MS1 (from IDA and SWATH™ spectra) and MS2 quantification (SWATH™) to assess the reproducibility of used techniques and robustness of our results. Most MUPs show highly similar fold changes regardless of the quantification strategy used. However, two proteoforms showed a higher upregulation in MS2-based SWATH™ quantification. A possible explanation is that MS1-based quantification in complex samples is strongly influenced by background ions such as co-eluting peptides. In SWATH™ quantification additional filtering, similar to Multiple Reaction Monitoring (MRM), increases selectivity and sensitivity. This results in a higher dynamic range and therefore a more accurate relative quantification.^40,54^ Additionally, MUP quantification relies on one or few proteotypic peptides per proteoform, and therefore if one peptide is differentially identified in MS1 and MS2 the impact on fold change computation is greater than for proteins whose quantification depends on multiple peptides.

There are several advantages and disadvantages with MS1 and MS2 techniques that need to be considered. Differences between both strategies are induced by the low number of peptides selected for quantification and general differences between the two methods used. MS1-based quantification strategies extract precursor mass intensities from complex chromatograms/MS spectra.^52^ One advantage of MS1-based approaches is the relative ease of data processing without requirement of a dedicated quantification run.^53^ However, the risk of extracting areas of unspecific interferences due to co-eluting peptides (which is a relevant issue in highly homologous proteins) is increased compared to approaches using additional selectivity filters (*e.g.* SWATH™ or MS2-based strategies in general).^40,54^ One advantage of MS2-based strategies is that highly similar precursor masses can more easily be distinguished on fragment level, especially in complex mixtures.^30,40^ Although mouse urine is not a complex matrix, the homology of MUPs requires analysis of precursor fragments. On the MS2-level this additional specificity enables a more robust quantification through identifying peptides not only based on their precursor mass but also based on their specific fragment mass fingerprints.^55,56^ Furthermore, background intensities and other unspecific signals are heavily reduced. Therefore, a better signal-to-noise ratio can be achieved for the extracted individual peak areas.

Here, we demonstrate the use of three different label-free quantification strategies for quantification of highly homologous proteins. Commonly, different quantitative methods show a strong correlation when peptides with a sufficiently high signal-to-noise ratio in the XICs are used.^57^ Fold changed computed from MS1 quantification using PEAKS® were different from the approaches shown in Fig. 4 because different peptides were used for protein identification (see Fig. 2) and quantification. Thus, only strategies that allow for run–run alignment were used and the same ion library was chosen for each approach. MS1-intensities and areas of proteotypic peptides were exported from IDA spectra using MasterView™ in PeakView® and from SWATH™ spectra using Skyline. Here, we used the same ion-library as for SWATH quantification to ensure high comparability between SWATH™ and MS1 approaches and to reduce the variation originating from the use of two independent runs for calculating correlation of MUP quantification results.^58^ We showed a good correlation for most proteins (see Fig. 4). However, we found that quantification of individual proteoforms is sometimes based on different unique peptides. This result can explain how identical MUP proteoforms represented by different unique peptides do not necessarily correlate quantitatively (see Fig. 3A and B). This result might be induced by partial modifications or missed cleavages during proteolytic digest due to differences of tryptic activity relative to total protein concentration in individual samples. Consequently, the formation of tryptic peptides might be more efficient in some of the samples leading to an increase of missed cleavages in others.

Finally, we investigated the relative urinary MUP composition to differentiate proteoforms that differ in their relative abundance. By comparing spectra intensities from SWATH™ data we confirmed previous assumptions that the urinary proteome is composed mainly of MUPs. We found that MUP derived peptides account for 85 ± 7% of the total MS intensity. Additionally, relative intensities were shown and A2CEK6 (*Mup*13), B8JI96 (*Mup*14) and A2AV72 (*Mup*6) were identified as being the most abundant MUP proteoforms. Using a label-free quantification strategy such as SWATH™ to describe protein compositions is challenging and has its limitations. This approach provides relative rather than absolute quantification. When attempting to assess the composition of the MUP proteoforms (high and low abundant MUPs), one cannot account for different peptide ionization efficiencies. Nonetheless, since MUP proteoforms are highly homologous, this constraint is less of a problem than with other proteins and we additionally accounted for the number of unique peptides per proteoform.

## Conclusions

MUPs are highly homologous at gene and protein levels, making it difficult to assess variation, even with state-of-the-art proteomic methods. Our study provides several important contributions towards the analysis of inter- and intra-individual variation in urinary MUPs of house mice. First, we provide evidence on protein level for 19 MUP proteoforms in a population of wild house mice, using identification of unique peptides by high-resolution Q-TOF mass spectrometry. We identified currently unreviewed MUP proteoforms, and we will submit protein sequences to SwissProt to increase the number of reviewed MUPs available for other research groups and thus promote identification of proteoform-specific effects in chemosignalling. Second, we found that male mice show an proteoform-specific pattern of MUP upregulation during social interactions in seminatural conditions. Two MUPs, B8JI96 (*Mup*14) and P11589 (*Mup*2), were significantly upregulated (normalized by protein concentration) in MS1 and MS2 modus, regardless of the database used. Third, we explored several methodological issues regarding MS analyses to assess the importance of normalization, databases, and reproducibility of MS approaches. In general, normalization by protein concentration and background normalization using proteins expressed at constant levels (SP+MUP database) yielded the most significant results. We found that MS1 and MS2 quantification strategies are generally highly reproducible if the same ion library is used. The use of such state-of-the-art proteomic techniques makes monitoring of specific protein changes possible. This is especially important for analysis of MUP family members that cannot be discriminated by genetic or antibody-mediated methods.
