# Supplementary material for: Regulation of highly homologous major urinary proteins in house mice quantified with label-free proteomic methods
Source: Mol Biosyst. 2016 Jul 18;12(10):3005–16. doi: 10.1039/c6mb00278a (PMC5166567; doi:10.1039/c6mb00278a)
Supplement: Supplementary file 1 [file MB-012-C6MB00278A-s001.pdf]

Electronic Supplementary Material (ESI) for Molecular BioSystems.
This journal is © The Royal Society of Chemistry 2016

Supplementary

Technical Reproducibility

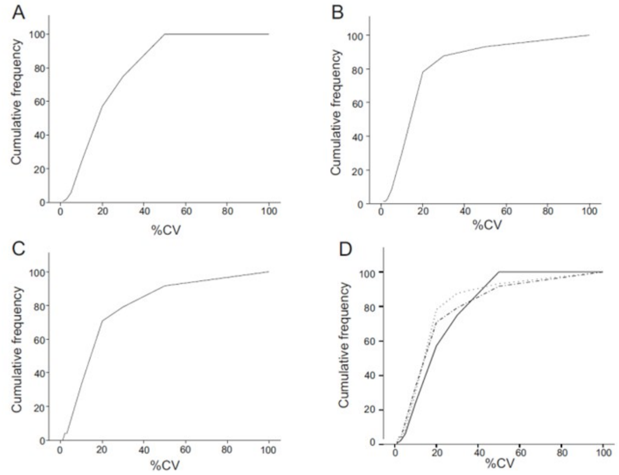

Figure S1: Technical reproducibility (triplicates) of SWATH™ measurements for urinary proteins. A) Transition level data, B) Peptide level data, C) Protein level data, D) all three graphs combined (solid line = Transition level data, dashed line = Peptide level data, dot-dashed line = Protein level data)

The technical reproducibility of a SWATH™ method for MUP quantification was shown in a pilot study using triplicates (Figure S1). All extracted peaks areas were hereby analyzed in triplicate and the coefficient of variation was assessed on fragment, peptide, and protein level. The data showed good reproducibility, so the quantitative experiments were performed using a maximum number of biological replicates rather than pooling individual samples and running technical replicates.

Urinary protein concentrations

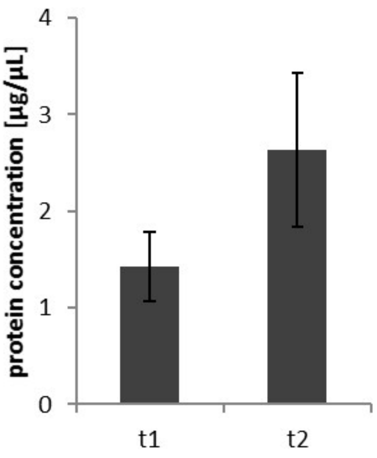

Figure S2: Plot of average urinary protein concentrations at both time points shows that protein concentrations are elevated at t2 due to increased social interactions in semi-natural housing.

The difference in protein concentrations between standard conditions (t1) and increased social interactions in seminatural conditions (t2) are shown in Figure S2. Commonly in proteomic studies the proteins of interest whose expression changes between experimental conditions are low abundant and the major part of the proteome is expressed without alteration. We decided to measure our samples without normalizing by protein concentration to reflect the increase of MUPs on MS level, because MUPs are the predominant proteins in male mouse urine. It is assumed that normalization by manual scale factors (protein concentrations) and TAS can account for different protein concentrations while data without normalization can also show proteins that are upregulated from t1 to t2 but do not have higher intensities relative to other MUPs at t2.
